# Assessing the Effect of Combined Humanization of Apolipoprotein E, Amyloid and Tau on Cognition and Alzheimer’s Disease Pathology

**DOI:** 10.1002/alz.092571

**Published:** 2025-01-03

**Authors:** Aya Arrar, Madison Longmuir, Kate M. Onuska, Beatriz G Muratori, Bryan Lung, Scheila Schmidt, Arash Salahinejad, Suzete M Cerutti, Lisa M Saksida, Timothy J Bussey, Tallulah S Andrews, Taylor W. Schmitz, Vania F Prado, Marco A.M Prado

**Affiliations:** ^1^ University of Western Ontario, London, ON Canada; ^2^ Robarts Research Institute, London, ON Canada; ^3^ Schulich Medicine and Dentistry, London, ON Canada; ^4^ Universidade Federal de São Paulo, São Paulo Brazil; ^5^ Schulich School of Medicine and Dentistry, London, ON Canada; ^6^ Western University, London, ON Canada; ^7^ Lawson Health Research Institute, London, ON Canada

## Abstract

**Background:**

Apolipoprotein E (ApoE) exhibits isoform‐specific interactions with Alzheimer’s disease (AD)‐related pathology. In comparison with the more common ApoE3 isoform, ApoE4 promotes amyloid‐β (Aβ) deposition, enhances tau‐mediated neurodegeneration and inflammation. However, the lack of appropriate preclinical models has limited the ability to evaluate the potential synergistic effect of Aβ, tau and ApoE on cognition and disease progression. To overcome this, we developed new mouse models harbouring humanized variants of the *ApoE* genotypes (*ApoE3 or ApoE4*), *hApp* (*App^NL^* or *App^NL‐F^*) and *hMAPT* (tau) and evaluated whether the interaction among these AD‐related factors influence cognition, brain structure, and pathological load.

**Method:**

An array of biochemical and imaging techniques including single‐nuclei (sn)RNA sequencing, magnetic resonance imaging (MRI), immunofluorescence microscopy, and Western blots were performed. Attentional demand was assessed, using the cross‐species Continuous Performance Task, an automated touchscreen test.

**Result:**

Preliminary results indicate that App^NL‐F^ (ApoE3 or ApoE4), but not App^NL^ (ApoE3 or ApoE4) mice presented plaque pathology and insoluble Aβ. Moreover, increased levels of insoluble Aβ, plaque burden, plaque size and neuroinflammation were observed in the cortex of App^NL‐F^ ApoE4 mice in comparison to App^NL‐F^ ApoE3 at 9 and 12 months of age. At 16 months of age App^NL‐F^ ApoE3 and ApoE4 mice showed similar levels of insoluble Aβ. *In vivo* 9.4T MRI analyses indicated reduced grey matter volume in fronto‐cortical regions of App^NL‐F^ ApoE4 mice when compared to controls. snRNA sequencing also indicated a reduction of excitatory neurons in the cortex and upregulation of gliosis related genes and astrocytic populations at 9 months in App^NL‐F^ ApoE4 mice. Attentional deficits were observed on the CPT as early as 6 months of age in App^NL‐F^ ApoE4 mice that were maintained at 9 and 12 months of age.

**Conclusion:**

Results from this project provide new insights into how amyloid, ApoE, and tau conspire to trigger and accelerate disease progression. We show that amyloid interacts with both ApoE3 and ApoE4 to drive degeneration, but in that the rate of this progression is faster in the ApoE4 background. Our findings also demonstrate that cognitive deficits are detectable much earlier than expected, in putative pre‐plaque stages of disease.

